# Alzheimer’s Disease-Associated Pathology in a Transgenic Mouse Model Results in Altered Voiding Function

**DOI:** 10.1093/geroni/igaa057.386

**Published:** 2020-12-16

**Authors:** Cara Hardy, Dawn Rosenberg, Ramalakshmi Ramasamy, Xiangyou Hu, Phillip Smith

**Affiliations:** 1 UConn Health, Farmington, Connecticut, United States; 2 UConn Health, Farminton, Connecticut, United States

## Abstract

Alzheimer’s disease (AD) is a devastating disorder primarily affecting older adults and is the most common neurodegenerative disease in the US. More than one in three AD patients experience AD-associated urinary dysfunction (ADUD), which directly contributes to their institutionalization. While ADUD has been clinically regarded as a result of poor cognitive control over urinary function, the physiology underlying loss of urinary control remains unknown. We hypothesize that beta-amyloidosis in the CNS results in pathologic changes in urinary structure and function. Male and female Tg-APP/PS1DE9 mice were used before plaque deposition (4-6 months) and after plaque accumulation (8-10 months) and compared to their WT littermates. Pressure-flow cystometry was conducted under urethane anesthesia to assess urinary performance at the level of the autonomic nervous system in the absence of cortical control. Pharmacomyography was performed on bladder strips to determine tissue-level changes in the absence of CNS input. In Tg-APP/PS1DE9 mice, plaque accumulation resulted in diminished volume sensitivity and decreased voiding efficiency. Pharmacologic studies showed aberrant drug responses, altered cholinergic signaling, and decreased resilience of tissue longevity after plaque accumulation. Based on our findings, we conclude that the AD-related pathology of Aβ accumulation results in a distinct urinary phenotype in our model, analogous to the ADUD observed in AD patients. Establishing and expanding models of ADUD to other mouse models of AD-associated pathology may improve the efficacy of treating ADUD and increase quality of life for patients and their caregivers.

